# Contrast-enhanced magnetic resonance imaging for the detection of ruptured coronary plaques in patients with acute myocardial infarction

**DOI:** 10.1371/journal.pone.0188292

**Published:** 2017-11-30

**Authors:** Christian H. P. Jansen, Divaka Perera, Andrea J. Wiethoff, Alkystis Phinikaridou, Reza M. Razavi, Aldo Rinaldi, Mike S. Marber, Gerald F. Greil, Eike Nagel, David Maintz, Simon Redwood, Rene M. Botnar, Marcus R. Makowski

**Affiliations:** 1 King’s College London, Division of Imaging Sciences and Biomedical Engineering, London, United Kingdom; 2 BHF Centre of Excellence, London, United Kingdom; 3 NIHR Biomedical Research Centre and King’s College London, London, United Kingdom; 4 Cardiovascular Centre, Guy’s and St. Thomas’ Hospital, London, United Kingdom; 5 Philips Healthcare, Guildford, United Kingdom; 6 Wellcome Trust and EPSRC Medical Engineering Center, London, United Kingdom; 7 Department of Radiology, University Muenster, Muenster, Germany; 8 Pontificia Universidad Católica de Chile, Escuela de Ingeniería, Santiago, Chile; 9 Department of Radiology, Charité, Berlin, Germany; University of Messina, ITALY

## Abstract

**Purpose:**

X-ray coronary angiography (XCA) is the current gold standard for the assessment of lumen encroaching coronary stenosis but XCA does not allow for early detection of rupture-prone vulnerable plaques, which are thought to be the precursor lesions of most acute myocardial infarctions (AMI) and sudden death. The aim of this study was to investigate the potential of delayed contrast-enhanced magnetic resonance coronary vessel wall imaging (CE-MRCVI) for the detection of culprit lesions in the coronary arteries.

**Methods:**

16 patients (13 male, age 61.9±8.6 years) presenting with sub-acute MI underwent CE-MRCVI within 24-72h prior to invasive XCA. CE-MRCVI was performed using a T1-weighted 3D gradient echo inversion recovery sequence (3D IR TFE) 40±4 minutes following the administration of 0.2 mmol/kg gadolinium-diethylenetriamine-pentaacetic acid (DTPA) on a 3T MRI scanner equipped with a 32-channel cardiac coil.

**Results:**

14 patients were found to have culprit lesions (7x LAD, 1xLCX, 6xRCA) as identified by XCA. Quantitative CE-MRCVI correctly identified the culprit lesion location with a sensitivity of 79% and excluded culprit lesion formation with a specificity of 99%. The contrast to noise ratio (CNR) of culprit lesions (9.7±4.1) significantly exceeded CNR values of segments without culprit lesions (2.9±1.9, p<0.001).

**Conclusion:**

CE-MRCVI allows the selective visualization of culprit lesions in patients immediately after myocardial infarction (MI). The pronounced contrast uptake in ruptured plaques may represent a surrogate biomarker of plaque activity and/or vulnerability.

## Introduction

Despite improvements in prevention, diagnosis and treatment, cardiovascular disease remains the leading cause of morbidity and mortality in Western industrialized nations and in developing countries. Whereas atherosclerosis alone is rarely fatal, sudden luminal thrombosis, superimposed on a ruptured or eroded atherosclerotic plaque, precipitates life threatening clinical events such as acute coronary syndromes and stroke [1–3]. Plaques thought to cause luminal thrombosis are referred to as vulnerable plaques. Histologically these types of plaques in most cases are so-called thin-cap fibroatheromas (TCFA). TCFAs are characterized by a large lipid or necrotic core separated from the coronary arterial lumen by a thin membrane cap [4]. Although various clinical studies using conventional invasive coronary angiography, intra-vascular ultrasound (IVUS), and cardiac computed tomography[5–7] have confirmed a strong relationship between atherosclerotic disease burden and risk for adverse events, there is no conclusive evidence that individual plaque assessment improves the prediction of acute coronary event risk compared to established risk factors, such as the extent and severity of coronary artery disease [8,9].

Because of its noninvasiveness, excellent soft-tissue contrast and ability to visualize the coronary lumen and vessel wall, magnetic resonance imaging (MRI) is a very promising imaging modality to assess coronary lumen integrity[10,11], atherosclerotic disease burden[12–21], plaque activity and composition[22–27]. Compared to native coronary MR vessel wall imaging[28,29], contrast-enhanced MR coronary vessel wall imaging (CE-MRCVI) is faster and potentially more robust, offering morphological as well as functional assessment of atherosclerotic plaque formation[30,31].

The aim of this study was to investigate the potential of CE-MRCVI for the characterization of culprit lesions in patients with high prevalence and known plaque vulnerability prior to invasive assessment and interventional treatment.

## Methods

### Study population

The study was approved by the local research ethics committee (Guy’s NHS Research Ethics Committee London, UK, Study No. 08/H0802/101). Written informed consent was obtained from all patients before inclusion into the trial. Participants in this study were recruited between June 2008 and January 2010. Sixteen patients with acute myocardial infarction were prospectively enrolled within 72 hours (45.1±22.6 hours) after initial onset of symptoms, who were not eligible for primary percutaneous coronary intervention. The mean age of participants was 61.9 years with an age range from 46 to 75 years. This included patients with non-ST-elevation acute coronary syndromes and those who had received thrombolysis for ST-elevation myocardial infarction. The diagnosis of acute myocardial infarction was based on elevation of cardiac biomarkers with at least one of the following: Electrocardiogram changes indicative of novel ischemia or development of pathologic Q-waves, symptoms of ischemia. Patients which were eligible for primary percutaneous coronary intervention, patients with clinical or electrocardiogram evidence of ongoing ischemia, significant cardiac arrhythmia and/or heart failure (New York Heart Association IV) were not included. Additional exclusion criteria were: < eighteen years of age, patients unable to give consent, impaired renal function (glomerular filtration rate less than 30 ml per min) mental disorders, contraindications to iodine or gadolinium, claustrophobia, breastfeeding or pregnancy. Within 24h (median 4.5 h; range 1–26 h) prior to invasive x-ray coronary angiography, the majority of patients (n = 15) were scanned successfully. Patients received glycoprotein 2b/3a inhibitors or stenting during x-ray coronary angiography depending on the interventional cardiologist.

### Magnetic resonance imaging, X-ray coronary angiography and culprit lesion definition

All participants were investigated on a clinical 3T magnetic resonance imaging (MRI) scanner (Achieva, Philips Healthcare, Best, the Netherlands) in a supine position. The scanner was equipped with a specific cardiac coil (32 channels, InVivo Corporation, USA) in combination with a specific software package for cardiovascular imaging. Patients were monitored with a four-lead electrocardiogram, an oxygenation sensor to assess blood oxygen levels and a respiratory belt. Before the image acquisition was started diethylenetriamine-pentaacetic acid (0.2 mmol/kg DTPA, Magnevist, Bayer-Schering, Berlin, Germany) was administered via an intravenous access. First the coronary arteries were localized. Then a 3D T2-prepared fast GRE (gradient echo) sequence was acquired. In the next step the three point planscan tool was used to define acquire imaging planes parallel of the right and left coronary artery system. Subsequently a coronary magnetic resonance angiography (coronary MRA) was performed using a 3D T2prep fast GRE (TFE) sequence [32]. Imaging parameters of the sequence were: number of slices = 20, acquired in-plane resolution = 1.25 x 1.25 mm, reconstructed slice thickness = 1.5 mm (acquired: 3 mm), acquisition matrix = 256 x 256, field of view = 320 x 320 mm, acquisition window = 80 to 100 ms, flip angle = 20 degrees, repetition time = 5.5 ms, echo time 1.7 ms, and startup cycles = 5. Contrast enhanced (CE) imaging of the coronary artery wall was started 31 min (mean time 31.2 minutes, range 26.5 to 34.7 minutes) following the administration of diethylenetriamine-pentaacetic acid. A T1-weighted fat-suppressed (FS) 3D GRE inversion recovery (3D IR TFE) MR sequence was used. The main imaging parameters, including the orientation of the imaging sequence as well as the spatial resolution were identical to the initially performed coronary MRA. Differences existed regarding the flip angle = 30 degrees, repetition time = 5.7 ms, the echo time = 1.8 ms; additionally, an adiabatic (nonselective) inversion radiofrequency pulse was used instead of the T2 preparation pulse. The sequence was ECG-triggered and navigator-gated. The exact sequence design was also described in a previous publication[26]. The inversion time of the scan (median 230, range 180 to 280 ms) was specifically adjusted to minimize signal from the blood on a patient by patient basis. To achieve an optimal nulling off signal from the blood a region of interest was used on the look locker sequence to calculate the most precise inversion time. Based on the information available from the twelve lead electrocardiogram, the most likely affected coronary vessel system was scanned first. X-ray coronary angiography was performed using standard techniques with multiple projections. The culprit lesion was defined as the site with the smallest diameter [33].

### Analysis of magnetic resonance imaging and X-ray coronary angiography

**Fig 1:**
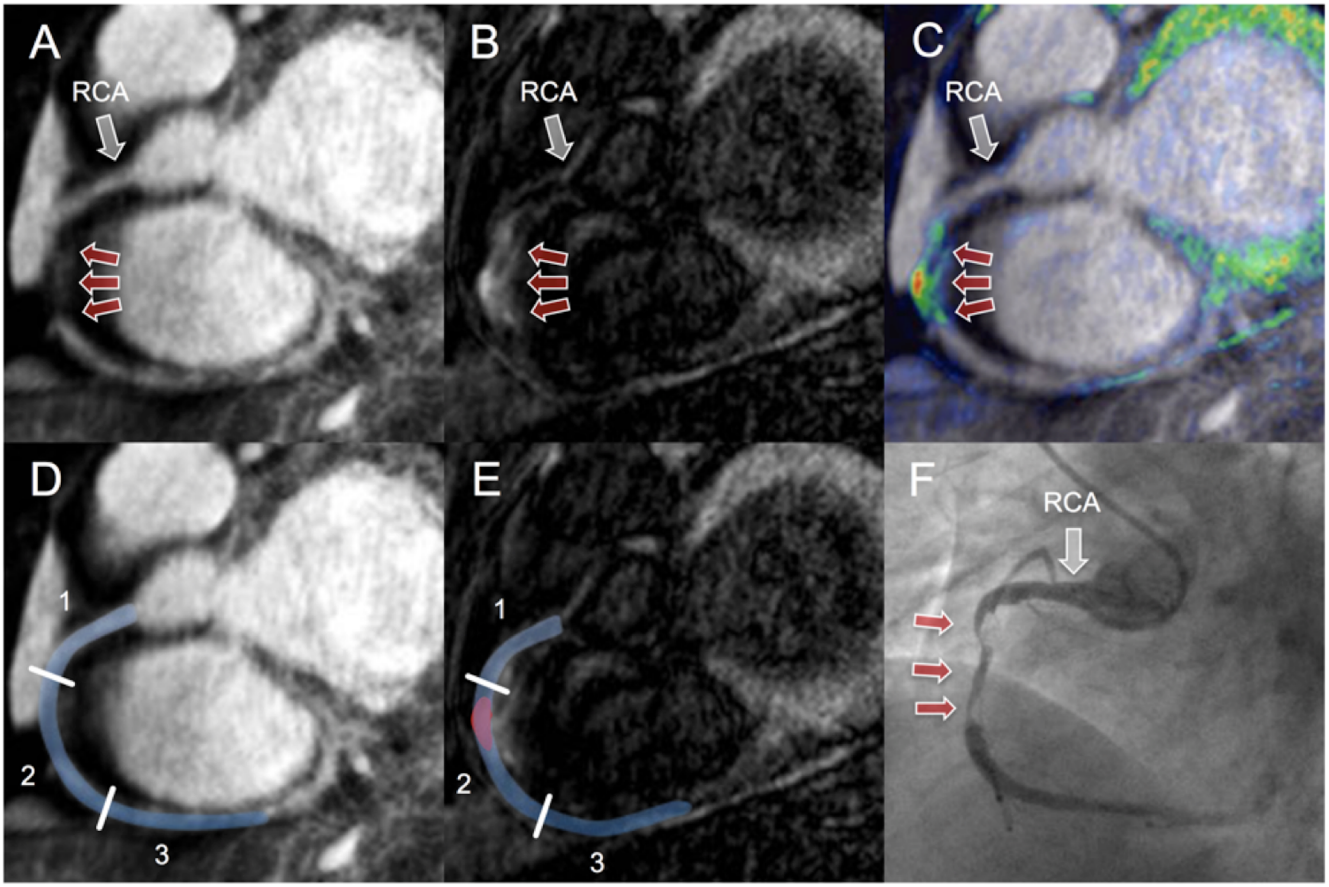
CE-MRCVI findings in a patient with troponin positive ACS in the RCA. Coronary MRA (A) of a 48-year-old male with troponin positive ACS showed decreased vessel lumen size of the mid RCA (red arrows). To highlight the relationship between CE-MRCVI (B) and morphology (A), images were fused in a way similar to PET/CT (C). CE-MRCVI displays high signal intensity (red arrows) within the mid RCA. Analysis of signal enhancement on CE-MRCVI (E, red) suggestive for culprit lesion yielded a contrast-to-noise ratio (CNR) of 12.7. Corresponding XCA (F) confirmed CE-MRCVI findings with mid RCA de-novo lesion (pre-treatment stenosis of 75–94%). MRA: magnetic resonance angiography, CE-MRCVI: contrast enhanced magnetic resonance coronary vessel wall imaging, PET/CT: positron emission tomography/computed tomography. CE-MRCVI:contrast enhanced magnetic resoance coronary vessel wall imaging, ACS: acute coronary syndrome, RCA: right coronary artery, MRA: magnetic resonance angiography, XCA: x-ray coronary angiography, PET/CT: positron emission tomography/computed tomography.

As described in previous publications a specific postprocessing tool (soapbubble) was used to reform eight data sets and to analyze the coronary magnetic resonance angiography as well as the contrast-enhanced images[34]. To compare magnetic resonance imaging to x-ray angiography a previously described eight segment model was used[26]. The coronary angiography was analyzed by two interventional cardiologists (DP, SR) with extensive experience. The length of the coronary artery vessel wall of each segment was quantified as performed in a previous study[32]. Coronary artery segments in which a stent was present as well as segments distal to stents were specifically excluded from the analysis. This was due to the inadequate suppression of signal from blood in these segments because of Faraday shielding effects and susceptibility artifacts. To visualize the colocalization of the coronary artery wall enhancement with regards to the course of the coronary arteries, the coronary magnetic resonance angiography and the contrast-enhanced images were automatically fused using a DICOM viewer (OsiriX, Geneva, Switzerland, Fig 1).

Findings were classified into three categories: 1^st^ “normal” coronary segments without visually apparent culprit lesion formation, 2^nd^ affected coronary segments with visually apparent culprit lesion formation and 3^rd^ segmented culprit lesion. Contrast-to-noise ratio (CNR) of “normal” coronary segments was determined by manual segmentation of the normal segment including coronary vessel wall and blood as described previously (Fig 1D) [30]. In case of visually apparent culprit lesion formation, affected coronary segment was manually segmented and affected coronary segment SNR including mean signal of blood, vessel wall and culprit lesion was calculated. Culprit lesion CNR was determined by manual segmentation of visually apparent culprit lesion area (Fig 1E). Noise (N) was determined in a ROI placed in the lung with the exclusion of areas with visible vascularization. Contrast-to-noise ratio (CNR) was measured between coronary (SI_coronary-vessel-wall_) and aortic blood signal. The CNR was calculated by the following formula: CNR = (SI _coronary-vessel-wall_ − SI_blood)_)/N.

Relative CNR values were calculated as ratio between visually apparent culprit lesion area, area of the entire affected coronary segment (including culprit lesion formation) or area of “normal” segments without culprit lesion and the averaged CNR of the corresponding entire left or right coronary system. To account for variation in signal intensity across the FOV due to spatial variation of the coil sensitivity an automatic correction for coil sensitivity (CLEAR) was applied. Relative CNR value measurements were repeated to calculate the intraobserver and interobserver variabilities. Two observers analyzed data of a subset of 10 patients in an independent manner, blinded to the angiography.

### Statistical analysis

The statistical analysis in this study was performed using *SAS 9*.*2 (SAS Institute Inc*., *Cary*, *USA*) and PASW Statistics software 18.0.0 (SPSS Inc, Chicago). Values were reported as mean ± with a 95% confidence interval (CI), mean ± standard deviation. If values were not normally distributed and they were reported as median ± range. For the comparison of continuous values a paired student’s t-test was performed. For the comparison of categorical values a Wilcoxon signed rank test was performed. P values lesser than 0.05 were considered significant. Per segment comparisons were performed, which were not independent of each other. To statistically correct for that, segments with and without culprit lesions, adjacent coronary segments of the same artery or coronary system were tested using repeated measures mixed linear models including compound symmetry *as working correlation matrix to account for multiple measurements*. *Regarding the comparison of contrast to noise ratio differences of the different groups mixed model with t-tests was applied*. Relative CNR value measurements were repeated to calculate the intraobserver and interobserver variabilities. Two observers analyzed data of a subset of ten patients in an independent manner and blinded to the angiography. To determine the cutoff values for CNRs (relative and absolute) between segments without culprit lesions and with culprit lesions, receiver-operating characteristics (ROC) curves were used. To determine the best cutoff value, the Youden-index was used.

## Results

### Patients characteristics

Table 1 summarizes the patient characteristics of the investigated study population. A total of 16 patients (13 men, age 61.9±8.6) with: 1) anterior STEMI (n = 7), 2) inferior STEMI (n = 7) and 3) NSTEMI (n = 2), were included in this study. At the time of inclusion all patients had no clinical or ECG evidence of ongoing ischemia. CMR scans were performed within 72 hours (median 45.2h, 8.3–70.8h) after onset of symptoms. Patients with anterior STEMI had a higher TIMI Risk Score (7.3 vs. 2.2, p = 0.027) and were older (age 66.8±8.0 vs. 58.5±8.0) as compared to patients with inferior STEMI. The incidence of failed thrombolysis (n = 7, thrombolysis for STEMI with ST resolution >90min) was identical in both groups.

**Table 1:** Baseline characteristics of patients with acute myocardial infarction. Characteristics of patients with acute myocardial infarction, including patients with anterior and inferior STEMI. Characteristics include typical risk factors, laboratory findings, TIMI risk score and GRACE risk score.

	Patient with acute myocardial infarction n = 16	Patients with anterior STEMI n = 7	Patients with inferior STEMI n = 7	p
Age (years)	61.3 ± 8.6	66.8 ± 8.0	58.5 ± 8.0	0.03
Male, n (%)	13 (81%)	5 (71%)	6 (86%)	ns
Weight (kg)	78.8 ± 13.9	75.6 ± 15.6	75.6 ± 11.9	ns
BMI	26.1 ± 3.1	25.5 ± 2.2	24.9 ± 3.4	ns
Median Risk factors, n	2	2	3	ns
Hypercholesterolaemia, n (%)	12 (69%)	5 (71%)	5 (71%)	ns
Hypertension	9 (56%)	6 (86%)	3 (43%)	ns
Diabetes mellitus	2 (13%)	2 (29%)	0 (2%)	ns
Smoking	8 (50%)	3 (43%)	4 (57%)	ns
Family history of CAD	7 (44%)	3 (43%)	3 (43%)	ns
Medical history of coronary artery stenting	1 (7%)	1 (17%)	0 (0%)	ns
ST-Segment Elevation Myocardial Infarction (STEMI)	14 (88%)	7 (100%)	7 (100%)	ns
Anterior STEMI	7 (44%)			
Failed thrombolysis	12 (75%)	6 (86%)	6 (86%)	ns
Median Blood pressure, mmHg				
Systolic, mmHg	115	120	105	ns
Diastolic mmHg	73	65	65	ns
Median Heart rate, bpm	69	75	67	ns
Laboratory findings				
Troponin T, ng/ml	2.0 (0.2–8.0)	1.0 (0.2–5.2)	2.1 (0.6–7.4)	ns
Leucocytes, 10E9	10.8 (5.8–35.0)	9.3 (6.1–15.4)	12.4 (11.7–18.1)	ns
C-reactive protein, mg/dl	9.0 (< 5–60)	7.5 (6.0–9.0)	8.0 (2.5–60.0)	ns
Platelets, 10E9	227 (160–653)	235 (160–328)	228 (168–653)	ns
TIMI Risk Score, %Death or MI	4.4 (1.6–23.4)	7.3 (2.2–23.4)	2.2 (1.6–7.3)	0.027
GRACE Risk Score, %Death or MI				
In-Hospital	18 (7–29)	18 (15–29)	19 (14–22)	ns
6 month follow up	31.0 (13–40)	31 (25–40)	34 (22–37)	ns

### Findings on x-ray coronary angiography

X-ray coronary angiography findings of the study population are summarized in Tables 2 and 3. XCA was performed within the following day after CE-MRCVI (median 4.5 h; range 1–26 h). In 14 of 16 patients a culprit lesion was identified by XCA and located in the left anterior descending artery (LAD, n = 7), left coronary artery (LCX, n = 2) and right coronary artery (RCA, n = 5) (Table 3). In one patient (Table 3—Patient 4) XCA revealed marked proximal ectasia of all the major coronary vessels without obvious culprit lesion. The posterior left ventricular artery (PLV) branch of the dominant RCA was occluded with a heavy thrombus load and filled retrograde from the left. The posterior descending artery (PDA) ostium also showed heavy thrombus burden, but flow was preserved (TIMI III flow). In the other patient (Table 3—Patient 13) with symptoms of ischemia, inferior ST segment elevation and positive cardiac necrosis markers, XCA showed non-stenotic coronary arteries after failed thrombolysis. Although late gadolinium MR images did not show a typical mid myocardial scarring, recent medical history and CRP increase up to 60 mg/l suggest myocarditis as the most likely cause for ECG changes and troponin rise.

**Table 2:** Angiographic findings in patients with acute myocardial infarction. Overview of patients with acute myocardial infarction, anterior STEMI, inferior STEMI and NSTEMI. Detailed description of angiographic findings including the degree of stenosis for the number of segments.

		Patient with acute myocardial infarction n = 16	Patients with anterior STEMI n = 7	Patients with inferior STEMI n = 7	Patients with NSTEMI n = 2
Vessel with disease, % (n)					
	"normal" vessels	14% (1/7) †	0% (0/7)	14% (1/7) †	0% (0/2)
	1 vessel disease	38% (6/16)	43% (3/7)	43% (3/7)	0% (0/2)
	2 vessel disease	44% (7/16)	43% (3/7)	29% (2/7)	100% (2/2)
	3 vessel disease	13% (2/16)	14% (1/7)	14% (1/7)	0% (0/2)
Angiographic findings, % (n)					
	"Normal" Segments	64.8% (83/128)	62.5% (35/56)	67.9 (38/56)	62.5 (10/16)
	Segments 1% to 49%	4.7% (6/128)	3.6% (2/56)	3.6% (2/56)	0.0 (0/16)
	Segments 50% to 74%	6.3% (8/128)	7.1% (4/56)	7.1% (4/56)	0.0 (0/16)
	Segments 75% to 94%	13.3% (17/128)	16.1% (9/56)	5.4% (3/56)	31.3 (5/16)
	Segments 95% to 99%	3.9% (5/128)	7.1% (4/56)	1.8% (1/56)	0.0 (0/16)
	Complete Occlusion	7.0% (9/128)	3.6% (2/56)	10.7% (6/56)	6.3 (1/16)
Culprit lesion location, % (n) #					
	RCA	43.8% (7/16)	0.0% (0/7)	85.7% (6/7)	50.0% (1/2)
	LAD	43.8% (7/16)	85.7% (6/7)	0.0% (0/7)	50.0% (1/2)
	LCX	6.3% (1/16)	14.3% (1/7)	0.0% (0/7)	0.0% (0/2)

Lesions were considered hemodynamically important when they caused ≥50% reduction of coronary luminal diameter

^†^ One patient with inferior STEMI and tenecteplase thrombolysis was diagnosed with non obstructed coronary arteries in XCA

**Table 3:** Diagnostic accuracy of CE-MRCVI culprit lesion location in comparison to XCA. This table gives an overview about the angiographic findings, ECG findings as well as the absolute and relative CNR value for each of the investigated patients.

	XCA	ECG	Visual Assessment	Absolute Values (CNR)			Relative CNR Values (%CNR)		
				Affected System*	Affected Segment	Culprit lesion	Affected System*	Affected Segment	Culprit lesion §
Patient 1	RCA 2	inferior STEMI	RCA 2	3.5 (3.0–4.1)	5.7	13.8	83% (70–96%)	134%	323%
Patient 2	RCA 2	inferior STEMI	RCA 2	2.4 (2.3–2.4)	2.6	6.5	97% (95–98%)	107%	264%
Patient 3	RCA 2	NSTEMI	RCA 2	1.7 (1.3–2.1)	4.9	12.7	63% (46–77%)	177%	460%
Patient 4	PLV/PDA†	inferior STEMI	none						
Patient 5	LCX 11	inferior STEMI	LCX 11	2.0 (0.6–2.8)	3.2	10.1	94% (29–131%)	151%	406%
Patient 6	LAD 6	anterior STEMI	LAD 6	1.6 (1.4–2.3)	3.2	5.3	82% (68–112%)	157%	236%
Patient 7	RCA 2	inferior STEMI	RCA 2	0.8 (0.6–0.9)	1.2	6.4	83% (68–98%)	134%	694%
Patient 8	RCA 2	inferior STEMI	RCA 2	0.6 (0.4–0.9)	2.6	6.5	49% (30–69%)	201%	495%
Patient 9	LAD 6	anterior STEMI	LAD 6	3.4 (2.9–5.3)	4.8	10.4	85% (74–135%)	121%	239%
Patient 10	LAD 7	NSTEMI	LAD 7	6.3 (4.3–6.6)	6.3	9.2	105% (72–111%)	106%	151%
Patient 11	LAD 7	anterior STEMI	LAD 7	5.3 (2.7–6.5)	5.7	7.9	104% (53–126%)	112%	138%
Patient 12	CX 11	anterior STEMI	CX 11	4.4 (3.9–8.2)	10.4	20.3	71% (63–131%)	165%	305%
Patient 13	none	inferior STEMI	none						
Patient 14	LAD 6	anterior STEMI	LAD 6	1.5 (1.1–1.9)	2.4	5.2	82% (58–106%)	135%	280%
Patient 15	LAD 7	anterior STEMI	LAD 7	4.1 (1.5–5.0)	4.8	11.5	105% (38–129%)	123%	313%
Patient 16	LAD 6	anterior STEMI	LAD 6	6.4 (2.3–8.9)	7.9	10.0	100% (36–139%)	124%	154%

* Affected coronary system (RCA or LCA) excluding segments with visually apparent culprit lesion

^†^ XCA showed heavy thrombus load in PLV branch and PDA (LV branch and PDA not included in 8 segment model for analysis)

^§^ Culprit lesion CNR in comaprison to all segments within affected coronary system (RCA or LCA) including affected segments

### CE-MRCVI

CE-MRCVI scans were successfully performed in all 16 subjects. In one patient (Table 3—Patient 16) with sub-acute STEMI, only CE-MRCVI of the left coronary artery (LCA) was obtained due to patient discomfort. A total of 119 (95% of available segments) were analyzed for coronary vessel wall enhancement.

### *CE-MRCVI—*Visual assessment

CE-MRCVI fused with coronary MRA (Fig 1) showed localized marked signal increase of the vessel wall in all 14 patients with culprit lesion formation on XCA (7 LAD, 2 LCX and 5 RCA). CE-MRCVI findings are summarized in Table 3. In the patient (Table 3—Patient 4) with marked proximal ectasia of all the major coronary vessels, CE-MRCVI did not show any circumscribed signal increase of the proximal, mid or distal RCA vessel wall. Based on XCA assessment, the proximal ectatic RCA segment in this patient was thought to be the most likely source of in-situ thrombus of the PLV branch and PDA ostium. These distal branches, which are not included in the 8-segment model, were not covered by the MRDTI scan.

### CE-MRCVI—Absolute CNR

**Fig 2:**
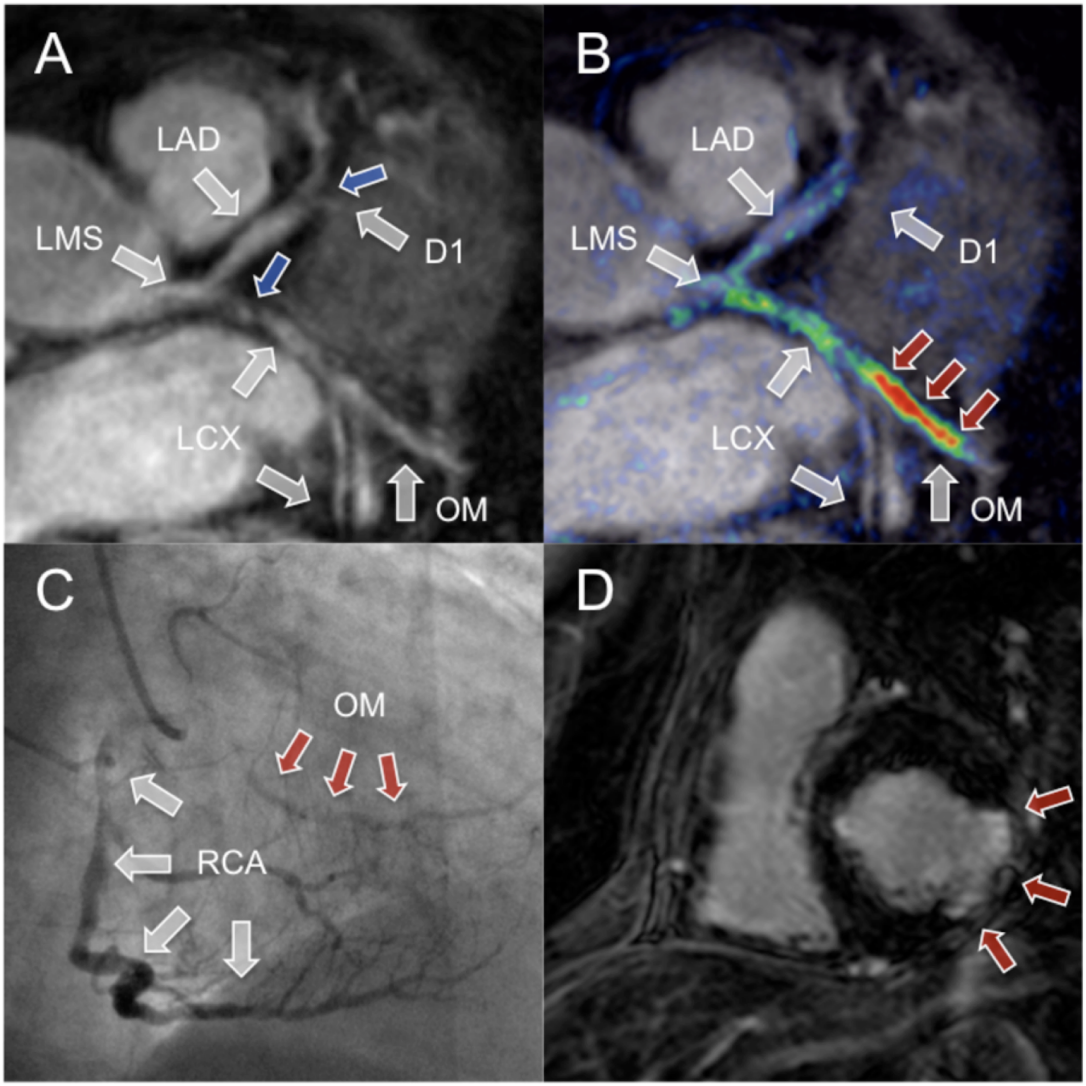
CE-MRCVI findings with troponin positive ACS in the LCX. Coronary MRA (A) with troponin positive ACS showed decreased vessel lumen size of the proximal LCX and first marginal branch (grey arrows). To highlight the relationship between and morphology (A) and CE-MRCVI, images were fused in a way similar to PET/CT (B). CE-MRCVI displays high signal intensity (red arrows) within the first marginal branch. Corresponding XCA (C) confirmed CE-MRCVI findings with mid LCX lesion. Short axis delayed enhancement scan shows predominantly transmural myocardial infarction of the mid-ventricular infero-lateral segment. MRA: magnetic resonance angiography, CE-MRCVI: contrast enhanced magnetic resonance coronary vessel wall imaging, PET/CT: positron emission tomography/computed tomography.

In patients shortly after MI signal intensity of coronary culprit lesion formation as measured by CNR (n = 14, CNR 9.7±4.1) was significantly increased compared to the CNR averaged over coronary segments with (n = 14, CNR 4.6±2.6, p<0.001) and without (n = 105, CNR 2.9±1.9, p<0.001) culprit lesion formation (Fig 2). Based on a ROC curve analysis, a CNR threshold of 6.3 was determined for the detection of culprit lesions in patients with sub-acute myocardial infarction (segment-wise analysis: area under the curve 0.967, sensitivity 86%, specificity 91%). Based on this cut-off value quantitative CE-MRCVI correctly identified the culprit lesion location in 12 out of 14 patients, but resulted in 9 false positive segments (1 RCA, 1 LMS, 2 LAD, 5 LCX). The majority of these misclassified segments (7 out of 9) occurred within the affected vascular system, resulting in 5 patients with multiple culprit lesions. All false positive segments in patents with confirmed culprit lesion (n = 8) had lower CNR values as compared to the corresponding true positive culprit lesion (CNR difference: 2.62, 1.1–13.4).

### CE-MRCVI—Relative CNR

**Fig 3:**
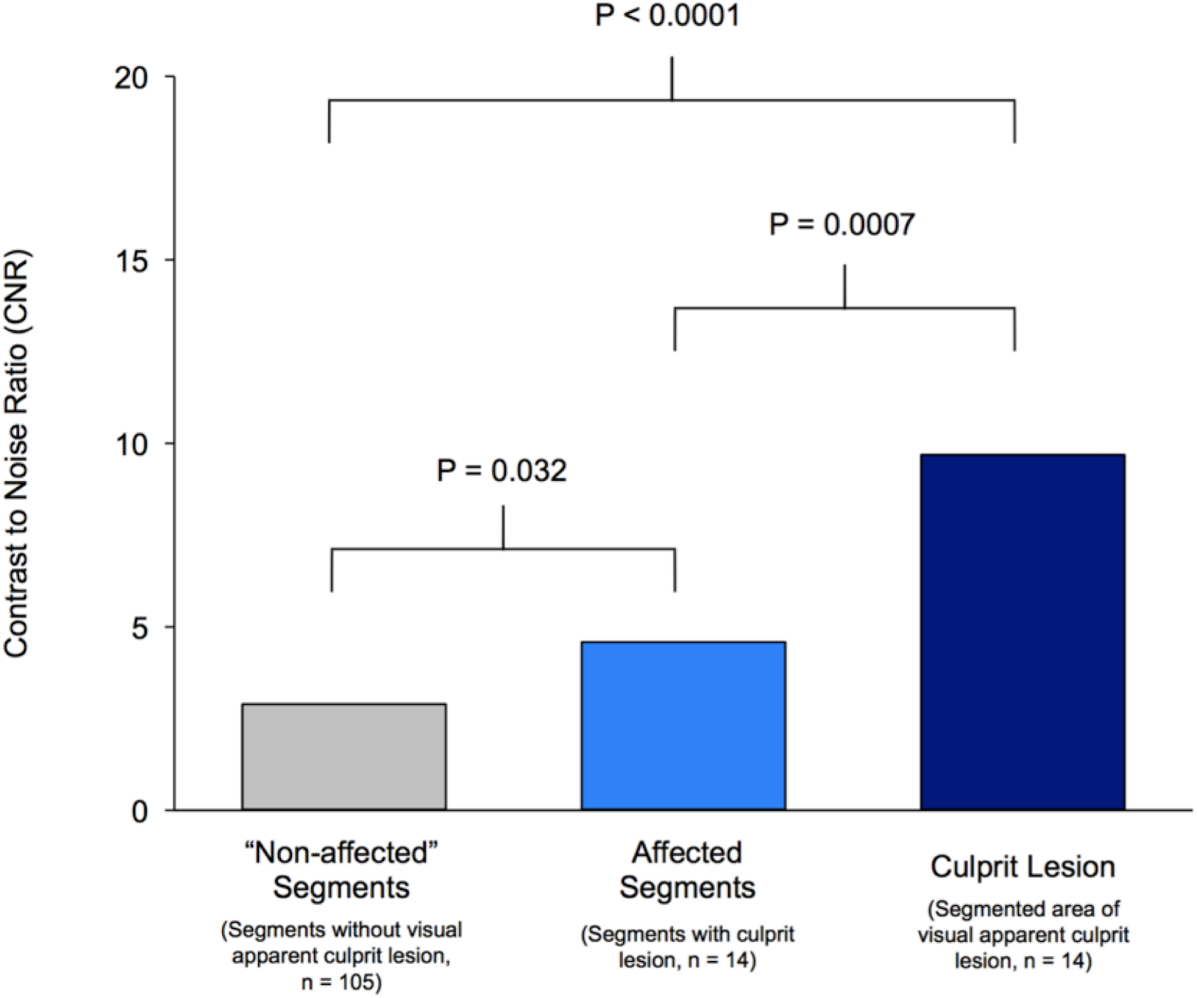
Contrast-to-noise ratio of culprit lesion and coronary vessel wall. Contrast to noise ratio (CNR) of segmented culprit lesion in comparison to segments with and without culprit lesion formation. Absolute CNR values of segmented culprit lesion area (Mean: 9.7, 95% CI: 7.6–11.9), segments with (Mean: 4.7, 95% CI: 3.4–6.0) and “normal” segments without visual apparent culprit lesion formation (Mean: 2.9, 95% CI: 2.5–3.3) were found to differ significantly (p<0.05).

**Fig 4:**
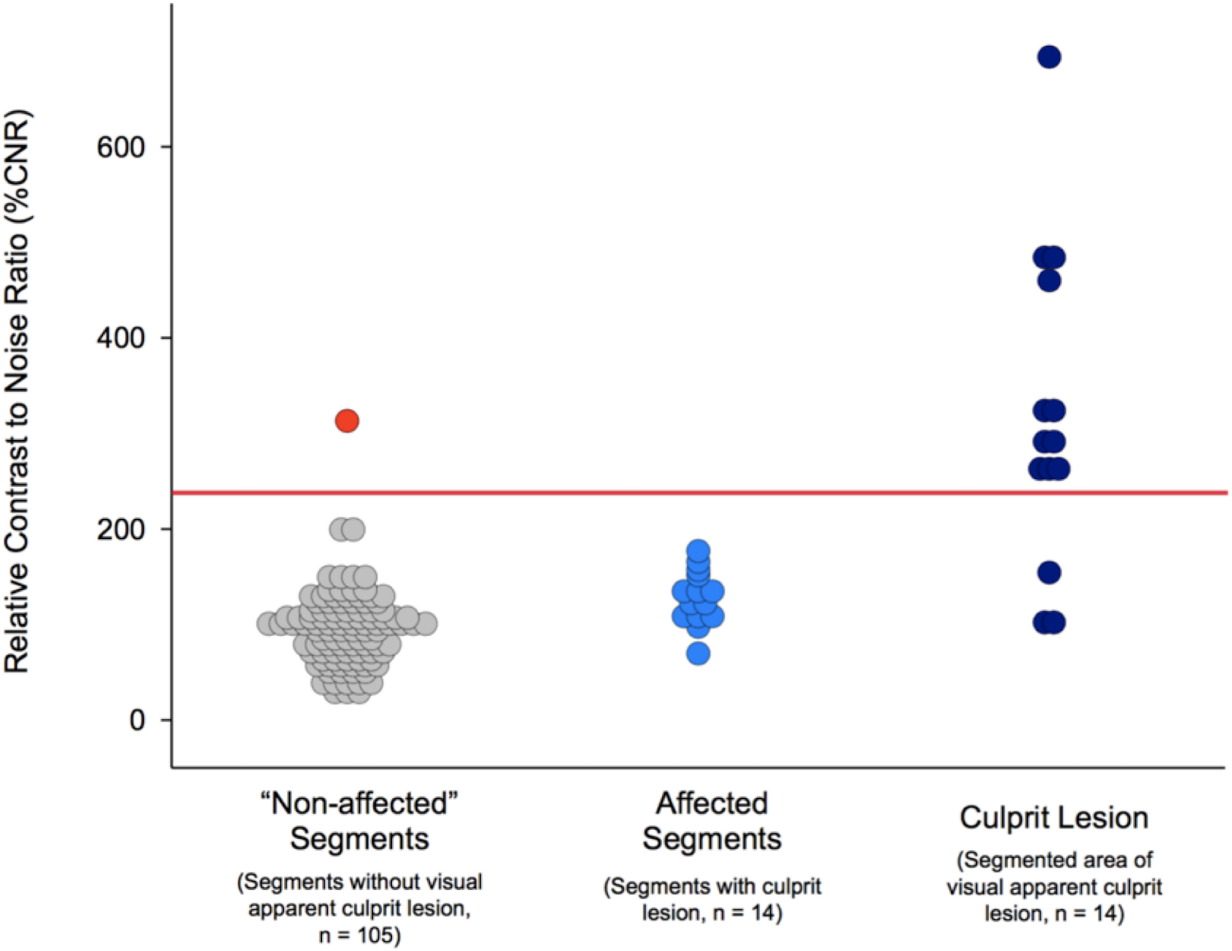
Relative contrast-to-noise ratio of culprit lesion and coronary vessel wall. Relative CNR values were calculated as ratio between visually apparent culprit lesion area, the affected coronary segment (including culprit lesion formation) or “normal” segments without apparent culprit lesion formation and the averaged CNR of the corresponding entire left or right coronary system. Relative CNR values of segmented culprit lesion area (Mean: 329%, 95% CI: 249–409%), affected coronary segments with culprit lesion (Mean: 130%, 95% CI: 116–144%), and “normal” segments without visual apparent culprit lesion formation (Mean: 96%, 95% CI: 88.54–103.46) were found to differ significantly (p<0.05). A relative CNR threshold of 230 (red line) resulted in a true positive detection and localization in 11 out of 14 patients and true exclusion in 104 out of 105 coronary segments. The false positive classification of the left main stem (red dot) was observed in a patient without culprit lesion in XCA (inferior STEMI).

Relative CNR values were calculated as ratio between individual segments or culprit lesion formation and the averaged CNR of all corresponding segments of either the left or right coronary system. Similar to the analysis of absolute CNR values, relative CNR values of culprit lesion formation (CNR 329±153%, 95% CI: 249–409%) were significantly increased as compared to the relative CNR averaged over coronary segments without (96±39%, 95% CI: 88.54–103.46, p<0.001) and with (130±27%, 95% CI: 116–144%, p<0.001) culprit lesion formation (Fig 3). Based on a ROC curve analysis a relative CNR threshold of 230 was determined for the detection of culprit lesion formation in patients with sub-acute myocardial infarction (segment-wise analysis: area under the curve 0.99, sensitivity 79%, specificity 99%). The use of a relative cut-off value resulted into true positive detection in 11 out of 14 coronary segments with culprit lesion formation and true negative classification of 104 of 105 segments without culprit lesion. The false positive classification of the left main stem (Fig 3, red dot) was observed in a patient without culprit lesion in XCA (Table 3 Patient 13, inferior STEMI). In comparison to absolute CNR, the use of relative CNR values yields higher overall specificity with slightly lower sensitivity (Fig 4). The Bland–Altman analyses demonstrated no significant differences between repeated enhancement measurements (-9%CNR for intraobserver error, p>0.05 and −11%CNR for interobserver error p>0.05). Intra-/interobserver agreements with 95% limits of agreement ranging from -35 to 18%CNR and -44 to 22%CNR were acceptable.

## Discussion

This study demonstrated the potential of contrast-enhanced magnetic resonance imaging (CE-MRCVI) for the detection of culprit lesions in patients with sub-acute myocardial infarction prior to invasive assessment and interventional treatment. CE-MRCVI in 14 patients with sub-acute myocardial infarction allowed for a direct visualization of culprit lesions in the LAD, LCX and RCA with high contrast relative to the blood pool and the surrounding tissue. Culprit lesion CNR significantly exceeded the corresponding values of segments with and without culprit lesion formation.

### Non contrast enhanced and contrast-enhanced MR imaging of coronary atherosclerosis, plaque burden and vascular remodeling

Different non-contrast enhanced and contrast-enhanced MR techniques can be used for the noninvasive and non-radiation associated characterization of coronary atherosclerosis.

Non-contrast enhanced MR vessel wall imaging (NCE-MRCVI) has shown promise for the visualization and quantification of coronary vessel wall thickness, vascular remodeling and plaque burden in previous studies [12–17,19,20]. Additionally, non-contrast enhanced coronary plaque imaging is useful for the identification of high intensity plaques (HIP) associated with intraplaque hemorrhage[24] and/or coronary thrombus [26].

CE-MRCVI based techniques can further add to NCE-MRCVI and together provide comprehensive characterization of coronary atherosclerosis. CE-MRCVI currently relies on the use of non targeted specific gadolinium based contrast agents and enables a morphological and functional (plaque activity) assessment of atherosclerotic plaques. Non-targeted gadolinium-based contrast agents are approved for clinical use and imaging techniques (LGE-MRI) for their visualization are relatively robust and typically used for imaging of myocardial infarction. Clinically approved non-specific gadolinium compounds rapidly extravasate into the vessel wall and enhance areas with either increased distribution volume and delayed clearance (typically fibrosis) or increased endothelial permeability and neovascularization (inflammation). Previous studies have demonstrated the potential of CE-MRCVI for the characterization of the coronary artery wall in patients with giant cell arteritis and Takayasu’s arteritis[35–38], sub-acute myocardial infarction [30] or coronary allograft vasculopathy in children[39]. In the context of atherosclerosis, serial MR imaging of coronary plaques[30] demonstrated a CNR increase of up to 40% during the acute phase of myocardial infarction.

The current study demonstrated that quantitative CE-MRCVI correctly identified the culprit lesion location with a sensitivity of 79% and excluded culprit lesion formation with a specificity of 99%. The contrast to noise ratio (CNR) of culprit lesions (9.7±4.1) significantly exceeded CNR values of segments without culprit lesions (2.9±1.9, p<0.001). The use of relative CNR values in this study yielded a higher overall specificity with slightly lower sensitivity, in comparison to absolute CNRs. Compared to previous studies[30] relative CNR values of culprit lesions were higher, with up to 230% for segments with culprit lesions. In contrast, in a previous study investigating the chronic “stable” phase 3 month after myocardial infarction only a moderate increase of 20% was measured. Whereas the latter finding is thought to be related to plaque volume and fibrosis, the comparatively higher contrast uptake in this study potentially reflects acute inflammatory changes. These inflammatory processes could represent the main contributor for increased coronary plaque contrast uptake due to an increased endothelial permeability and an increased level of angioneogenesis in the extracellular matrix of inflamed plaques in the sub-acute phase of myocardial infarction.

CE-MRCVI vessel wall imaging could therefore represent a noninvasive technique to 1) differentiate between stable and high-risk plaques in patients with coronary atherosclerosis and 2) to monitor disease activity with respect to acute inflammatory changes.

### Clinical potential of contrast-enhanced MR imaging of coronary atherosclerosis

With cardiovascular disease remaining the leading cause of morbidity and mortality in Western industrialized nations and in developing countries [40–45], strategies to prevent acute coronary events and their consequences are of high importance. The majority of acute coronary events are triggered by plaque rupture or erosion coinciding with vascular thrombosis [4,46]. Fibrous cap thickness (FCT) is one of the most important determinants of plaque vulnerability with TCFA recognized as a precursor lesion for plaque rupture [3,4]. The PROSPECT (Providing Regional Observations to Study Predictors of Events in the Coronary Tree) study demonstrated that TCFA is the highest risk plaque phenotype and the majority of event-related TCFA where characterized by a larger plaque burden and smaller lumen area [47]. Although TCFA are considered the highest risk plaque phenotype leading to myocardial infarction, many plaques rupture without clinical syndromes leading to progressive lumen obstruction and chronic ischemic heart disease [48]. Several imaging studies confirmed morphological plaque changes within a few month gaining or losing “vulnerable” plaque characteristics and demonstrated a high prevalence of TCFAs at various stages of disease in patients with CAD [49].

Although emerging imaging modalities like OCT have far surpassed the limits of the early days of angiography, the invasiveness and peri-procedural radiation exposure limit widespread clinical use for cardio-vascular risk assessment and treatment monitoring. Similarly, Multi Detector Computed Tomography (MDCT) and Positron Emission Tomography (PET) have made great progress in plaque assessment and both techniques have shown very promising results in identifying vulnerable plaque in patients with cardiovascular disease or acute coronary syndrome. Screening of larger populations or serial imaging after interventional or medical treatment is however severely hampered by the relatively high radiation exposure.

Because of its non-invasiveness, excellent soft-tissue contrast and ability to visualize the coronary vessel wall, MR is a very promising imaging modality. This study demonstrated that the information gained from CE-MRCVI has the potential to supplement current risk stratification with the ultimate goal to identify and monitor subsets of patients with pan-coronary vulnerability benefitting from a more aggressive systemic anti-inflammatory treatment rather than local or segmental interventional therapy. Despite the transitioning from a focus on individual lesions to atherosclerotic disease burden for coronary artery disease risk assessment[9], individual plaque assessment may proof useful in a subset of patients (e.g. proximal lesions with large soft plaques and luminal narrowing) to monitor plaque activity and response to medical treatment.

### Limitations

Due to ethical reasons, only patients not eligible for primary PCI were enrolled prospectively after sub-acute myocardial infarction, thus generalizability of our results is limited by selection bias with high probability of plaque erosion or rupture. Unlike target specific contrast agents which are currently restricted to preclinical imaging (e.g. elastin specific molecular probe[50]) or are limited by a prolonged timespan between contrast administration and imaging (e.g. ultra-small superparamagnetic particles of iron oxide for inflammation imaging), clinical approved and wide spread available non-specific compounds rapidly extravasate into the vessel wall and enhance areas with either fibrosis or increased endothelial permeability and neovascularization (inflammation). Although previous studies support inflammation as decisive contributor for increased contrast uptake within the culprit lesion, differentiation and individual contribution of plaque size, endothelial permeability and neovascularization remains challenging with nontargeted MR contrast agents. No comparison between in vivo MRI findings and invasive methods such as optical coherence tomography (OCT) or intravascular ultrasound (IVUS) were available as reference standard as part of this study.

## Conclusion

This study demonstrated that CE-MR vessel wall imaging has potential for the selective detection of culprit lesions in patients with acute myocardial infarction. The pronounced contrast uptake in ruptured plaques may represent a surrogate marker of plaque activity and/or vulnerability. Further studies are now warranted to investigate the clinical utility to characterize atherosclerotic plaque activity and vulnerability in a more heterogeneous group of patients with or without CAD.
